# Polygenic risk score for schizophrenia and structural brain connectivity in older age: A longitudinal connectome and tractography study

**DOI:** 10.1016/j.neuroimage.2018.08.075

**Published:** 2018-12

**Authors:** C. Alloza, S.R. Cox, M. Blesa Cábez, P. Redmond, H.C. Whalley, S.J. Ritchie, S. Muñoz Maniega, M. del C. Valdés Hernández, E.M. Tucker-Drob, S.M. Lawrie, J.M. Wardlaw, I.J. Deary, M.E. Bastin

**Affiliations:** a Division of Psychiatry, University of Edinburgh, Edinburgh, UK; b Centre for Cognitive Ageing and Cognitive Epidemiology, University of Edinburgh, Edinburgh, UK; c Department of Psychology, University of Edinburgh, Edinburgh, UK; d Scottish Imaging Network: A Platform for Scientific Excellence (SINAPSE) Collaboration, University of Edinburgh, Edinburgh, UK; e MRC Centre for Reproductive Health, University of Edinburgh, UK; f Centre for Clinical Brain Sciences, University of Edinburgh, Edinburgh, UK; g Department of Psychology, University of Texas, Austin, TX, USA

**Keywords:** Schizophrenia, Ageing, Structural connectivity, Longitudinal, Genetics

## Abstract

Higher polygenic risk score for schizophrenia (szPGRS) has been associated with lower cognitive function and might be a predictor of decline in brain structure in apparently healthy populations. Age-related declines in structural brain connectivity—measured using white matter diffusion MRI —are evident from cross-sectional data. Yet, it remains unclear how graph theoretical metrics of the structural connectome change over time, and whether szPGRS is associated with differences in ageing-related changes in human brain connectivity. Here, we studied a large, relatively healthy, same-year-of-birth, older age cohort over a period of 3 years (age ∼ 73 years, N = 731; age ∼76 years, N = 488). From their brain scans we derived tract-averaged fractional anisotropy (FA) and mean diffusivity (MD), and network topology properties. We investigated the cross-sectional and longitudinal associations between these structural brain variables and szPGRS. Higher szPGRS showed significant associations with longitudinal increases in MD in the splenium (β = 0.132, *p*_*FDR*_ = 0.040), arcuate (β = 0.291, *p*_*FDR*_ = 0.040), anterior thalamic radiations (β = 0.215, *p*_*FDR*_ = 0.040) and cingulum (β = 0.165, *p*_*FDR*_ = 0.040). Significant declines over time were observed in graph theory metrics for FA-weighted networks, such as mean edge weight (β = −0.039, *p*_*FDR*_ = 0.048) and strength (β = −0.027, *p*_*FDR*_ = 0.048). No significant associations were found between szPGRS and graph theory metrics. These results are consistent with the hypothesis that szPGRS confers risk for ageing-related degradation of some aspects of structural connectivity.

## Highlights

• Large longitudinal MRI study of brain structure in narrow age range older age cohort.
• Longitudinal changes occur in white matter tracts from age 73 to 76.
• Graph theory metrics also showed longitudinal reductions over that period.
• Microstructural changes in white matter correlate with changes in brain’s network.
• Higher genetic risk for schizophrenia is associated with changes in brain structure.

## 1 Introduction

Patients with schizophrenia show white matter impairments in post-mortem examinations and in *in vivo* studies using diffusion MRI (Harrison, 1999; Kubicki and Shenton, 2014). Less healthy brain white matter microstructure and the structural connectome have been associated with cognitive impairments in schizophrenia (Alloza et al., 2017, 2016; Kochunov et al., 2017; Yeo et al., 2016). Reports of less healthy water diffusion MRI parameters in schizophrenia are well documented; specifically, impairments are observed in the uncinate fasciculus, corpus callosum, cingulum and arcuate fasciculus (Burns et al., 2003; Ellison-Wright and Bullmore, 2009; Kelly et al., 2017; McIntosh et al., 2005). Likewise, healthy relatives who are at high risk of developing schizophrenia for genetic reasons also show white matter abnormalities in several tracts (Muñoz Maniega et al., 2008).

Graph theory segregation measures, such as clustering coefficient and modularity, have been reported to be altered in schizophrenia (Alexander-Bloch et al., 2010; Collin et al., 2013; van den Heuvel and Fornito, 2014; Zalesky et al., 2011), suggesting a more segregated pattern of network organization. Longer path lengths and reductions in communication efficiency between regions have also been found in patients diagnosed with schizophrenia, suggesting that schizophrenia may be characterised by reduced communication between distal brain regions (reviewed in van den Heuvel and Fornito, 2014). Graph theoretical studies have also reported small-world organization and reductions in integration and efficiency in unaffected relatives (Collin et al., 2014), indicating a genetic basis for schizophrenia. Despite the difficulties of coupling graph theory metrics and the underlying neurobiology, graph theory metrics have consistently shown associations with cognitive functions (Alloza et al., 2017; Collin et al., 2016; Li et al., 2009), symptoms (Collin et al., 2016; van den Heuvel and Fornito, 2014; Wang et al., 2012), heritability (Bohlken et al., 2014) and sensitivity to disease (Lynall et al., 2010; Rubinov et al., 2009), indicating that they do compute relevant properties of the brain's structure in this disorder.

Schizophrenia is both highly heritable and polygenic, with many common alleles of small effect, and increasing numbers of genome-wide significant loci being identified as sample sizes increase (Hilker et al., 2018; International Schizophrenia Consortium et al., 2009; Schizophrenia Working Group of the Psychiatric Genomics Consortium, 2014). The largest twin study in schizophrenia to date estimated its heritability to be 79%, and the proband-wise concordance rate in monozygotic twins to be 33%, suggesting that illness vulnerability is partly, but not exclusively, due to genetic factors (Hilker et al., 2018). The latest schizophrenia genome wide association study (GWAS) included a meta-analysis with 40675 cases and 64643 controls; it identified 179 independent genome-wide significant single nucleotide polymorphisms (SNPs) (P < 5 × 10^−8^) associated with a diagnosis of schizophrenia (Pardiñas et al., 2018; Schizophrenia Working Group of the Psychiatric Genomics Consortium, 2014). Summary statistics from large-scale GWAS allow the degree of genetic liability for a heritable trait (in this case, schizophrenia) to be estimated in healthy subjects outside the population in which the original GWAS was conducted (Van der Auwera et al., 2017, 2015).

In addition to schizophrenia, advancing age is associated with an increased risk for neurodegeneration, including white matter microstructure (Aboitiz et al., 1992; Cox et al., 2016; Hasan et al., 2010; Kochunov et al., 2015, 2012; 2011; Lebel et al., 2012; Marner et al., 2003; Meier-Ruge et al., 1992; Peters, 2002; Westlye et al., 2010) and cognitive decline (Deary et al., 2009; Verhaeghen and Salthouse, 1997). Therefore, identifying the determinants of the degree to which an individual experiences these cognitive and brain declines with age is a high priority (Corley et al., 2018). In ageing populations, a higher genetic risk for schizophrenia has been associated with both poorer cognitive function and with less healthy white matter (McIntosh et al., 2013; Muñoz Maniega et al., 2008). However, the neurobiological underpinnings of these apparent differences in cognitive ageing have not yet been fully explored.

Thus far, only a small number of studies have analysed the relationship between polygenic risk score for schizophrenia (szPGRS) and neuroimaging biomarkers in healthy and patient samples (Alloza et al., 2017; Birnbaum and Weinberger, 2013; McIntosh et al., 2013; Ritchie et al., 2017; Van der Auwera et al., 2015; Whalley et al., 2015). Emerging evidence suggests that higher szPGRS might be a predictor of accelerated decline in brain microstructure in older age. Ritchie et al. (2017) reported a significant longitudinal association between szPGRS and a general factor of tract-averaged mean diffusivity (MD; β = −0.120, SE = 0.059, *p* = 0.041, where a negative association indicates a link with unhealthy ageing), using a threshold of *p* = 1.00 derived from a previous GWAS (Schizophrenia Working Group of the Psychiatric Genomics Consortium, 2014) and 3-year change in the same dataset presented here (the Lothian Birth Cohort, 1936; LBC 1936). This nominal association did not, however, survive correction for multiple comparisons. Nevertheless, the largest published schizophrenia GWAS to date has improved considerably its predictive power (Pardiñas et al., 2018) and fibre tracking and analysis have been updated significantly to improve tract segmentation in this sample (Muñoz Maniega et al., 2017). These developments allow a more thorough investigation of the relationships between genetic risk for schizophrenia and structural brain connectivity in this ageing population than has hitherto been possible.

In this paper, we therefore investigated the hypothesis that szPGRS relates to white matter microstructure in older age by first mapping the trajectories of water diffusion MRI parameters (using fractional anisotropy (FA) and mean diffusivity (MD)) measured in twelve major tracts and the topological properties of FA-weighted networks in the LBC1936 across a three-year period. Secondly, we investigated the effect of szPGRS on these longitudinal tractography and connectome microstructural properties. We hypothesised that there would be a decline in brain connectivity (water diffusion MRI parameters and connectome network properties) over time, and that lower initial levels and steeper declines in these brain parameters would be found in those subjects with higher genetic liability for schizophrenia. As an additional analysis, we also investigated the hypothesis that higher szPGRS is associated with a steeper decline in cognition via change in white matter structure in older age.

## 2 Methods

### 2.1 Participants

The LBC1936 study (Deary et al., 2012, 2007; Taylor et al., 2018) provides longitudinal data on cognitive and brain ageing. The cohort comprises participants of the Scottish Mental Survey of 1947 (*SMS 1947, n* = 70,805) in which most Scottish schoolchildren born in 1936 sat the Moray House Test Number 12 at ∼11 years of age (Scottish Council for Research in Education, 1949). Most participants resided in the Edinburgh and Lothian regions of Scotland at recruitment age ∼70 years. The sample has been repeatedly tested in later life with participants undergoing detailed medical, physical, and psycho-social assessments, including a brain MRI examination (Wardlaw et al., 2011). The first testing wave took place at a mean age of 69.53 years (SD, 0.83 years) in 2004–2007 (n = 1,091, 543 females); the second testing wave took place at a mean age of 72.49 years (SD, 0.71 years) in 2007–2010 (n = 866, 418 females); and the third testing wave took place at a mean age of 76.25 years (SD, 0.68 years) in 2011–2014 (n = 697, 337 females). The data in the present report come from the second and third waves of the study at which points structural brain imaging was performed. A total of 731 participants (343 females) agreed to undergo brain imaging at a mean age of 72.68 years (SD, 0.72 years), and 488 (228 females) at a mean age of 76.38 years (SD, 0.65 years), none of whom were known to have schizophrenia. Only one participant was diagnosed with bipolar disorder. However, the data indicated that this participant was not an outlier ( ±2.5 SD for all brain imaging measures) and therefore, this subject was not excluded from the analysis. The study was approved by the Multi-Centre Research Ethics Committee for Scotland (MREC/01/0/56), the Lothian Research Ethics Committee (LREC/2003/2/29) and the Scotland A Research Ethics Committee (07/MRE00/58). All participants completed written informed consent forms before any cognitive, MRI, or other measurements were taken.

### 2.2 Scan acquisition

All structural and diffusion MRI data were acquired using a GE Signa Horizon HDx 1.5 T clinical scanner (General Electric, Milwaukee, WI, USA) using a self-shielding gradient set with maximum gradient strength of 33 mT m^−1^, and eight-channel head array coil. Diffusion-weighted echo-planar volumes (*b* = 1000 s mm^−2^) were acquired in 64 non-collinear directions, along with seven T_2_-weighted volumes (*b* = 0 s mm^−2^). Each volume comprised seventy-two contiguous axial 2-mm-thick slices acquired with 2 × 2 mm in-plane resolution. Repetition and echo times were 16.5 s and 95.5 m s respectively. A 3D T_1_-weighted inversion recovery-prepared fast spoiled gradient-echo (FSPGR) volume was also acquired in the coronal plane with 160 contiguous slices and 1.3 mm^3^ voxel dimensions. Full details of the imaging protocol are available (Wardlaw et al., 2011). The scanner underwent a major upgrade just prior to the first wave of imaging and was regulated continuously within a tight quality control environment across the duration of the study; all scans were acquired with the same imaging protocol and scanner software platform (Wardlaw et al., 2011) throughout.

### 2.3 Image processing

Each 3D T_1_-weighted FSPGR volume was parcellated into 85 cortical (Desikan et al., 2006) regions-of-interest (ROI) using FreeSurfer (http://surfer.nmr.mgh.harvard.edu), which comprised 34 cortical ROIs and eight sub-cortical ROIs per hemisphere, plus the brainstem. Segmentations were visually checked, then used to construct grey and white matter masks for use in network construction and to constrain the tractography output as described below. Using tools provided by the FDT package in FSL (http://fsl.fmrib.ox.ac.uk/fsl), the diffusion MRI data were pre-processed to reduce systematic imaging distortions and bulk subject motion artefacts by afﬁne registration of all subsequent EP volumes to the ﬁrst T_2_-weighted EP volume (Jenkinson and Smith, 2001). Skull stripping and brain extraction were performed on the registered T_2_-weighted EP volumes and applied to the mean diffusivity/fractional anisotropy (MD/FA) volumes calculated by DTIFIT in each subject (Basser and Pierpaoli, 1996; Smith, 2002). The neuroanatomical ROIs determined by FreeSurfer were then aligned from 3D T_1_-weighted volume to diffusion space using a cross-modal nonlinear registration method. As a ﬁrst step, linear registration was used to initialize the alignment of each brain-extracted FA volume to the corresponding FreeSurfer extracted 3D T_1_-weighted brain volume using a mutual information cost function and an afﬁne transform with 12 degrees of freedom (Jenkinson and Smith, 2001). Following this initialization, a nonlinear deformation ﬁeld based method (FNIRT) was used to reﬁne local alignment (Andersson et al., 2007). FreeSurfer segmentations and anatomical labels were then aligned to diffusion space using nearest neighbour interpolation.

### 2.4 Tractography

Whole-brain probabilistic tractography was performed using FSL's BedpostX/ProbTrackX algorithm (Behrens et al., 2007). Probability density functions, which describe the uncertainty in the principal directions of water diffusion, were computed using a two-ﬁbre model per voxel (Behrens et al., 2007). Twelve major tracts were identified in each participant using probabilistic neighbourhood tractography (PNT), as implemented in the TractoR package for fibre tracking and analysis (http://www.tractor-mri.org.uk/(Clayden et al., 2011; Muñoz Maniega et al., 2017); PNT is an automatic tract segmentation method that has shown good reproducibility (Clayden et al., 2009b). This technique optimizes the choice of seed point placement for tractography by estimating the best matching tract from a series of candidate tracts generated from a neighbourhood of voxels (typically 7 × 7 × 7) placed around a central voxel transferred from standard to native space against a reference tract that was derived from a group of healthy volunteers aged 25–64 years (Muñoz Maniega et al., 2017). The topological tract model was also used to reject any false positive connections, thereby significantly improving tract segmentation (Clayden et al., 2009a). The seed point best matching each tract to the reference was determined in this manner and probabilistic white matter tracts masks were reconstructed by sampling 5000 streamlines. All segmented white matter tracts were visually assessed to ensure they were an anatomically accurate representation of the fasciculi-of-interest. The resulting tractography masks were applied to the MD/FA volumes of each participant; this permitted tract-specific mean values of FA and MD, weighted by the connection probability, to be obtained for each tract in each subject. The twelve tracts segmented were the genu and splenium of corpus callosum, and bilateral cingulum, anterior thalamic radiations (ATR), arcuate, uncinate and inferior longitudinal fasciculi.

### 2.5 Structural connectome

Using the probability density functions generated from BedpostX/ProbTractX, streamlines were then constructed by sampling from these distributions during a tracking process that involved all white matter voxels using 100 Markov Chain Monte Carlo iterations with a ﬁxed step size of 0.5 mm between successive points. Tracking was initiated from all white matter voxels (Buchanan et al., 2014) in two collinear directions until terminated by the following stopping criteria designed to minimize the amount of anatomically implausible streamlines: (i) exceeding a curvature threshold of 70°; (ii) entering a voxel with FA below 0.1 (Verstraete et al., 2011); (iii) entering an extra-cerebral voxel; (iv) exceeding 200 mm in length; and (v) exceeding a distance ratio metric of 10. The distance ratio metric (Bullitt et al., 2003), excludes implausibly tortuous streamlines. For instance, a streamline with a total path length 10 times longer than the distance between end points was considered to be invalid. The values of the curvature, anisotropy and distance ratio metric constraints were set empirically and informed by visual assessment of the resulting streamlines.

### 2.6 Network construction

FA-weighted networks were constructed by recording the mean FA value along streamlines connecting all 85 ROI (network node) pairs from the default FreeSurfer cortical (Desikan et al., 2006) and subcortical regions. The endpoint of a streamline was considered to be the ﬁrst grey matter ROI encountered when tracking from the seed location. The average brain network across the cohort was determined by including those connections which occurred in more than 2/3 of the participants at baseline (de Reus and van den Heuvel, 2013). This baseline network mask was then propagated to the second wave of connectivity matrices. Organizational properties of the different networks were then obtained using the brain connectivity toolbox (www.brain-connectivity-toolbox.net). For each FA-weighted connectivity matrix for the average network, five global network measures were computed, namely mean edge weight (mean value of FA across the network), density (the fraction of present connections to possible connections), strength (the average sum of weights per node), clustering coefﬁcient (fraction of triangles around a node) and global efﬁciency (the average of the inverse shortest path length).

### 2.7 Polygenic risk score calculation

The majority of participants provided blood samples at the first testing wave (age 70 years) that were used to extract DNA for the genetic analyses. To measure single-nucleotide polymorphisms (SNPs) we used the Illumina 610-Quadv1 whole-genome SNP array; measurements were completed at the Wellcome Trust Clinical Research Facility Genetics Core, Western General Hospital, Edinburgh (https://www.wtcrf.ed.ac.uk). Stringent quality control analyses were applied to the genotype data which resulted in 549692 of the 599011 SNPs on the Illumina 610 chip being retained in 3511 individuals (2115 females). The sample collection, quality control and genotyping process is described in greater detail elsewhere and non-European individuals were carefully excluded from the current analysis (Davies et al., 2011). PGRS summarise the small effects across all SNPs that contribute to the genetic liability of a phenotype (in this case, schizophrenia). The out-of-sample validation of the capacity of szPGRS to predict onset of schizophrenia has been reported to explain 24.43% (the estimate assumes a population risk of 1%) of the variance in liability (Pardiñas et al., 2018). szPGRS were created for all individuals with suitable genotype data; only genotypes passing stringent quality control were used in analyses. szPGRS were estimated using the recent summary data from a GWAS of schizophrenia comprising a meta-analysis of two studies (Pardiñas et al., 2018; Schizophrenia Working Group of the Psychiatric Genomics Consortium, 2014), which included 40675 cases and 64643 controls. szPGRS were estimated using the PRSice software package according to previously described protocols (Euesden et al., 2015), with linkage disequilibrium and distance thresholds for clumping of r^2^ = 0.2 and within a 250 kb window. Five scores were created for each individual using SNPs selected according to the significance of their association with the phenotype at nominal p-value thresholds of 0.01, 0.05, 0.1, 0.5 and 1.0 (all SNPs). Our primary analyses used scores generated from a list of SNPs with a GWAS training set of p ≤ 1.0 threshold as recommended previously in order to allow replication by other studies and to maximise the potential predictive capacity (Ware et al., 2017). However, results at p ≤ 0.1 and p ≤ 0.5 thresholds are presented in Supplementary Material Tables 3 and 4 Four multidimensional scaling factors (estimated from SNP data) were entered into the models as additional ‘nuisance’ covariates to control for population stratification, along with age. These multidimensional scaling factors have been previously identified to be adequate for accounting for population structure in this sample (Davies et al., 2011).

### 2.8 Statistical analyses

First, age-related changes for white matter tract MD/FA values and global graph theory measures were calculated using linear mixed models for those participants who completed both assessments. The package used for the linear mixed models was 'nlme' (Pinheiro et al., 2018) in R and standardised betas were reported. Age in days at the time of MRI acquisition and sex were entered as fixed effects and participant as a random effect. Moreover, for each connectivity metric, residuals were calculated from a linear regression predicting each metric from density (fraction of present connections to possible connections), and these were used in all analyses. This is because several global graph theory metrics depend on density and comparisons at constant density allow differences related to the topological reorganization of links to be assessed longitudinally. The use of graph theory to study network topology is a valuable framework while also being a challenging task. For instance, the number of nodes (N) or network's degree (*k*) will influence the computation of global theory metrics (see Brain Connectivity Toolbox for a detailed description of metrics: https://sites.google.com/site/bctnet). Therefore, comparing networks with different N or *k* can yield spurious results (Wijk et al., 2010). Instead of restraining all networks to a fixed *k* parameter, we chose to control each subject's graph theory measure for edge density. Therefore, models presented below compute density as a fixed effect for each graph metric. This allowed us to compare metrics longitudinally independently of their differences in density.

**Fig. 1:**
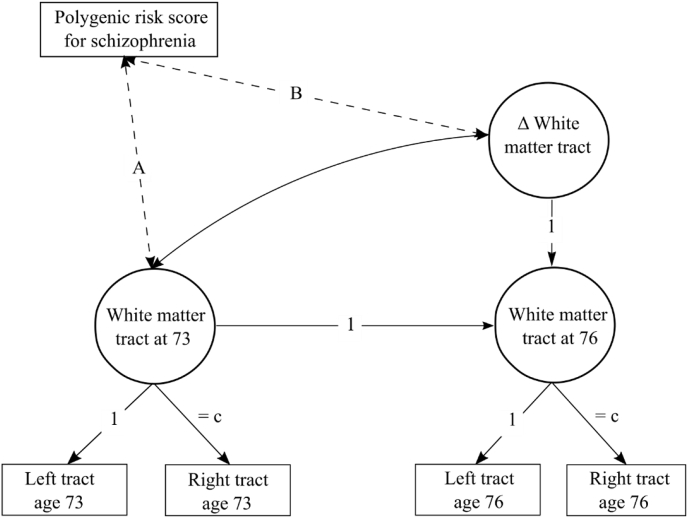
Diagram of the structural equation model (SEM) for white matter connectivity. A separate model was applied to each white matter tract (FA and MD) and each graph theory measure. Water diffusion and graph theory metrics were measured at baseline (age 73) and follow-up (age 76). From each individual bilateral white matter tract, a latent score was calculated for FA and MD. For callosal tracts and graph theory metrics a latent score was derived after the manifest variable was corrected for scaled age at scanning and sex. From these models, a latent change score variable was calculated for each model (Δ Connectivity). Relation between baseline FA/MD/graph theory measures and polygenic risk score for schizophrenia (szPGRS) is indicated by path A; path B represents the association between change in white matter FA/MD/graph theory measures and szPGRS. For all bilateral tracts, we further constrained equality of the factor loading of the left hemisphere (c). szPGRS was corrected for sex and population stratification while water diffusion MRI and graph theory measures at the manifest level were corrected for scaled age at scanning and sex at each time point within the model (paths not shown). Note that graph theory metrics were corrected for density outside the SEM model.

We then estimated a structural equation model (SEM) for each white matter tract MD/FA values and global graph theory measures. We estimated a separate model for each MRI metric (density-corrected network metric or white matter tract MD/FA value), which were set as the dependent variable in each model. Latent change score models (McArdle, 2009) were used to assess associations of szPGRS with the cross-sectional (baseline level, age ∼73 years) and longitudinal change (73–76 years) in diffusion MRI parameters. Latent scores were derived from bilateral white matter tracts. We constrained the loadings for left and right tracts across waves to be equal (i.e. the left loadings were equal across waves and independent of right loadings) (Persson et al., 2014). For interhemispheric white matter tracts (genu and splenium) and graph theory metrics, we used a single indicator model (Gollwitzer et al., 2014). Fig. 1 shows a simplified diagram of the SEM framework. Within the model, each brain imaging measure was adjusted for its respective age in days at the time of scanning and sex at the manifest level, while szPGRS was adjusted for sex and population stratification components. Due to the apparent association between schizophrenia and cardiovascular disease (Curkendall et al., 2004), we adjusted the linear mixed models and latent change score models for high blood pressure at each time point in order to reject the hypothesis that higher cardiovascular risk may contribute to a steeper decline in diffusion MRI parameters over time. For each model, we tested the association at the brain baseline level and change with szPGRS. SEM was performed using the package 'lavaan' (Rosseel, 2012) in R with full-information maximum likelihood estimation to use all data available.

**Fig. 2:**
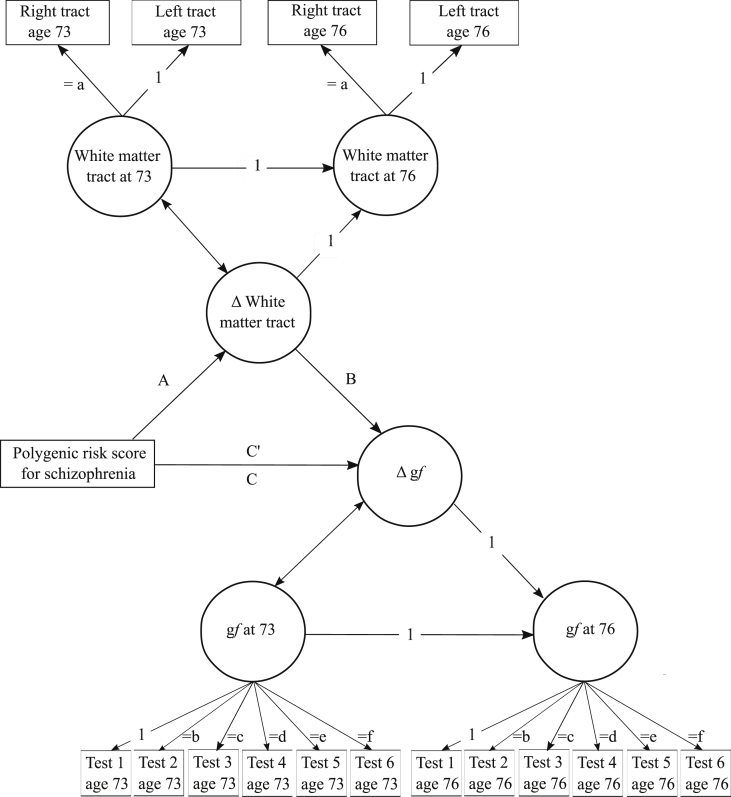
Diagram of the mediation model. The SEM model for white matter connectivity has been already described in Fig. 1. From each individual cognitive test, a latent score was calculated for general fluid intelligence (g_*f*_). From this model, a latent change score variable was calculated (Δ g_*f*_). Relation between polygenic risk score for schizophrenia (szPGRS) and change in white matter connectivity is indicated by path A; path B represents the association between change in white matter and change in g_*f*_. Path C represents the association between szPGRS and change in g_*f*_. C′ denotes the effect of szPGRS on change in g_*f*_ when change in white matter connectivity is taken into account in the model.

As an additional analysis, we examined the hypothesis that higher szPGRS was associated with a steeper decline in cognition via change in white matter structure. We used SEM in the ‘lavaan’ package (Rosseel, 2012) with full-information maximum likelihood estimation to derived a latent score of general fluid intelligence (g_*f*_) for each wave from six non-verbal tests of cognitive function from the Weschler Adult Intelligence Scale III^UK^ (Wechsler, 1955): matrix reasoning (non-verbal reasoning), block design (constructional ability), symbol search and digit symbol (processing speed), letter number sequencing and digit span backwards (working memory). Within the model, each cognitive test was adjusted for age in days at the time of assessment and sex at the manifest level. We constrained the loadings for each individual raw score across waves (i.e. equal loadings for matrix reasoning at baseline and follow-up). Beyond the analyses of szPGRS to the mediator (A path), to test whether the mediation (change from path C to C′) was statistically significant (*p*_*FDR*_ < 0.05), we tested whether the direct path of szPGRS to g_*f*_ (path C) and the indirect path from the mediator to g_*f*_ (path B) were significant. Fig. 2 shows a simplified diagram of the model that was used to examine this hypothesis. All significance (*p*) values (α = 0.05) were corrected for multiple comparisons using false discovery rate (FDR, *p*_*FDR*_) (Benjamini and Hochberg, 1995).

## 3 Results

Descriptive statistics, valid sample sizes after quality controls and longitudinal change for each brain imaging measure are provided in Table 1. At baseline, seven hundred and thirty-one subjects met the inclusion criteria with a mean age at MRI scanning of 72.73 (SD 0.72) years. At follow-up, four hundred eighty-eight subjects with a mean age at MRI of 76.43 (SD 0.65) years were scanned. Baseline data (age 73) on the structural connectome have already been published elsewhere (Wiseman et al., 2018). Descriptive statistics of cognitive tests and health conditions are presented in Supplementary Material Tables 1 and 2.

**Table 1:** Descriptive statistics for bilaterally averaged white matter water diffusion MRI parameters and graph theory metrics across both waves (age 73 and 76 years).

	*n*	Age 73	*n*	Age 76	*Overlapping sample*	*β*	*SE*	*p*_*FDR*_
Age in years (SD)	731	72.73 (0.72)	488	76.43 (0.65)				
Females (%)	731	46.92	488	46.72				
Polygenic risk for schizophrenia	640	−6.4 × 10^−4^ (0.2 × 10^−4^)						
White matter tracts								
FA								
Genu (SD)	633	0.376 (0.047)	457	0.375 (0.044)	415	−0.027	0.024	0.392
Splenium (SD)	652	0.508 (0.067)	458	0.504 (0.071)	427	−0.056	0.021	0.019*
Arcuate (SD)	616	0.425 (0.035)	439	0.422 (0.036)	397	−0.062	0.016	<0.001*
ATR (SD)	641	0.329 (0.030)	444	0.333 (0.030)	410	0.056	0.022	0.019*
Cingulum (SD)	631	0.424 (0.044)	457	0.425 (0.043)	413	−0.014	0.023	0.541
Uncinate (SD)	606	0.322 (0.028)	420	0.331 (0.028)	383	0.117	0.024	<0.001*
Inferior longitudinal fasciculus (SD)	662	0.379 (0.042)	463	0.380 (0.045)	437	−0.018	0.016	0.489
MD								
Genu (SD)	633	798.55 (79.17)	457	854.05 (87.01)	415	0.333	0.023	<0.001*
Splenium (SD)	652	816.76 (130.66)	458	864.22 (174.86)	427	0.171	0.023	<0.001*
Arcuate (SD)	616	653.02 (48.44)	439	691.62 (54.86)	397	0.377	0.014	<0.001*
ATR (SD)	641	747.45 (58.28)	444	792.59 (67.58)	410	0.361	0.021	<0.001*
Cingulum (SD)	631	630.39 (39.06)	457	668.07 (39.95)	413	0.452	0.020	<0.001*
Uncinate (SD)	606	763.01 (46.80)	420	795.68 (52.19)	383	0.345	0.019	<0.001*
Inferior longitudinal fasciculus (SD)	662	767.84 (80.42)	463	816.80 (111.64)	437	0.279	0.023	<0.001*
Network connectivity measures								
Mean edge weight (SD)	534	0.379 (0.020)	416	0.380 (0.019)	335	−0.039	0.017	0.048*
Strength (SD)	534	8.554 (0.719)	416	8.704 (0.628)	335	−0.027	0.011	0.048*
Global efficiency (SD)	534	0.242 (0.015	416	0.244 (0.013)	335	−0.027	0.016	0.120
Clustering coefficient (SD)	534	0.249 (0.015)	416	0.252 (0.014)	335	−0.001	0.016	0.935

*Note:* SD: Standard deviation, FA: fractional anisotropy, MD: mean diffusivity, beta: standardised estimates from the linear mixed models, SE: standard error. ILF: inferior longitudinal fasciculus. Asterisks represent significance from the linear mixed models (*p*_*FDR*_ < 0.05).

### 3.1 Longitudinal changes in brain structural connectivity

#### 3.1.1 White matter FA

**Fig. 3:**
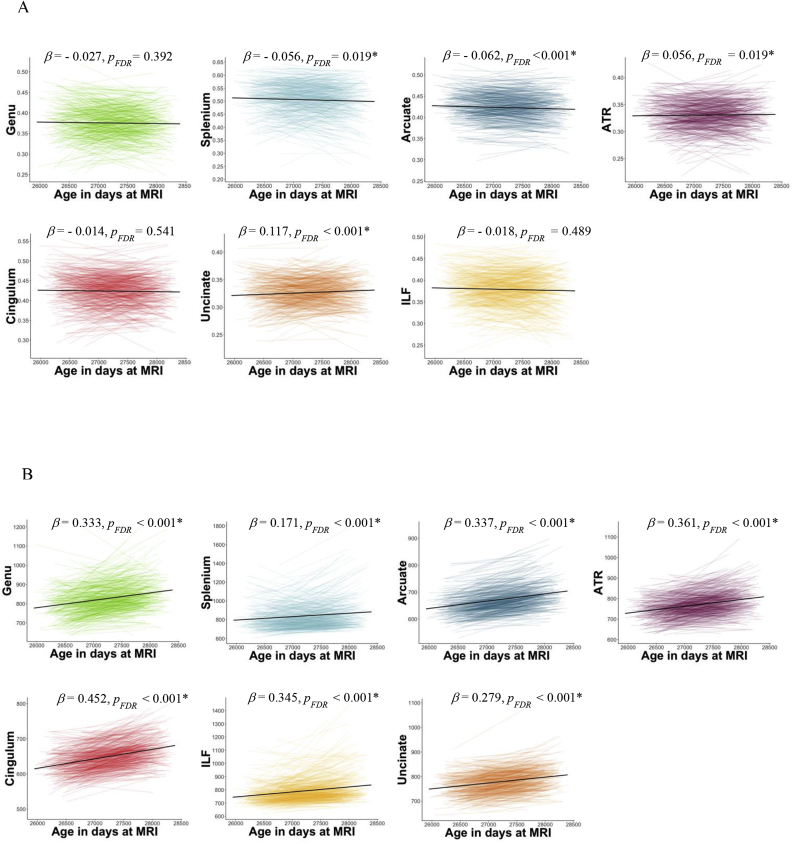
Trajectories of water diffusion MRI parameters over time. Each colour represents a different fibre for FA (plot A) and MD (plot B). The *x-*axis represents age in days at MRI scanning. The black line denotes linear regression. ATR = Anterior thalamic radiations; ILF = Inferior longitudinal fasciculus. Beta: standardised estimates from the linear mixed models. Asterisks represent significance from the linear mixed models (*p*_*FDR*_ < 0.05).

Results of the linear mixed models for FA are presented in Table 1 and Fig. 3A. Significant longitudinal reductions in FA were found for the splenium (β = −0.056, SE = 0.021, *p*_*FDR*_ = 0.019) and arcuate fasciculus (β = −0.062, SE = 0.016, *p*_*FDR*_ < 0.001). The genu (β = −0.027, SE = 0.024, *p* = 0.280), cingulum (β = −0.014, SE = 0.023, *p* = 0.541) and inferior longitudinal fasciculus (β = −0.018, SE = 0.016, *p* = 0.420) showed a non-significant decline over time (*p*_*FDR*_ > 0.05). Two white matter tracts showed significant longitudinal increases in FA, specifically the anterior thalamic radiations (ATR; β = 0.056, SE = 0.022, *p*_*FDR*_ = 0.019) and uncinate fasciculus (β = 0.117, SE = 0.024, *p*_*FDR*_ < 0.001). Sex had a significant effect on the FA of the splenium (β_sex_ = 0.111, SE = 0.036, *p*_*FDR*_ = 0.007), cingulum (β_sex_ = −0.093, SE = 0.035, *p*_*FDR*_ = 0.019) and inferior longitudinal fasciculus (β_sex_ = 0.142, SE = 0.035, *p*_*FDR*_ < 0.001). Positive effects (β_sex_) represent higher FA values in females compared to males, whereas negative effects represent higher FA values in males compared to females. As an additional analysis we tested for blood pressure effects; however, we did not find any significant effect of higher blood pressure on the longitudinal change of FA for any white matter tract (*p*_*FDR*_ > 0.05).

#### 3.1.2 White matter MD

Results of the linear mixed models for MD are presented in Table 1 and Fig. 3B. Significant longitudinal increases in MD were found for genu (β = 0.333, SE = 0.023, *p*_*FDR*_ < 0.001), splenium (β = 0.171, SE = 0.023, *p*_*FDR*_ < 0.001), arcuate (β = 0.377, SE = 0.014, *p*_*FDR*_ < 0.001), ATR (β = 0.361, SE = 0.021, *p*_*FDR*_ < 0.001), cingulum (β = 0.452, SE = 0.020, *p*_*FDR*_ < 0.001), uncinate (β = 0.345, SE = 0.019, *p*_*FDR*_ < 0.001) and inferior longitudinal fasciculus (β = 0.279, SE = 0.023, *p*_*FDR*_ < 0.001). Sex had a significant effect on the MD of the genu (β_sex_ = −0.117, SE = 0.033, *p*_*FDR*_ = 0.001), arcuate (β_sex_ = 0.116, SE = 0.036, *p*_*FDR*_ = 0.003), cingulum (β_sex_ = 0.127, SE = 0.032, *p*_*FDR*_ < 0.001) and inferior longitudinal fasciculus (β_sex_ = −0.079, SE = 0.033, *p*_*FDR*_ = 0.027). Positive effects (β_sex_) represent higher MD values in females compared to males, whereas negative effects represent higher MD values in males compared to females. Higher blood pressure was not significantly associated with longitudinal change in MD for any white matter tract (*p*_*FDR*_ > 0.05).

#### 3.1.3 Graph theory metrics

**Fig. 4:**
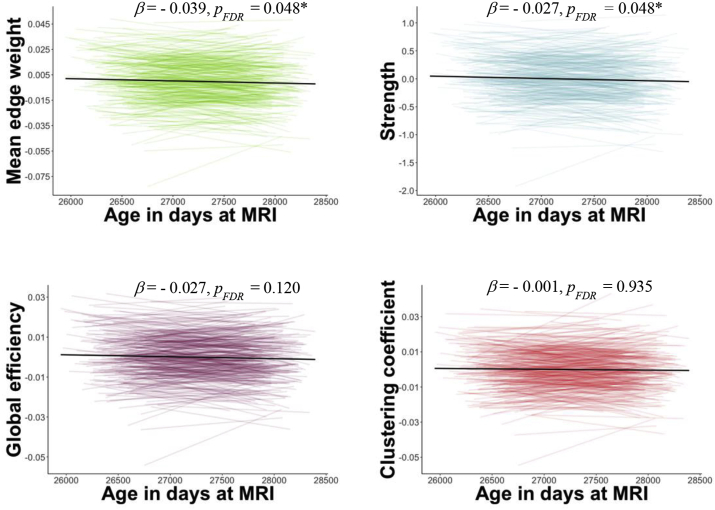
Trajectories of graph theory metrics between age 73 and 76 years. Plotted are residuals for each participant from the regression of the graph metric as the dependent variable and density and sex as the predictor variables. The *x-*axis represents age in days at MRI scanning. The black line represents linear regression. Beta: standardised estimates from the linear mixed models. Asterisks represent significance from the linear mixed models (*p*_*FDR*_ < 0.05).

Results of the linear mixed models for graph theory are presented in Fig. 4 and Table 1. There were longitudinal decreases in most graph theory metrics across all subjects. For instance, mean edge weight (β = −0.039, SE = 0.017, *p*_*FDR*_ = 0.048) and strength (β = −0.027, SE = 0.011, *p*_*FDR*_ = 0.048) declined significantly between waves. Global efficiency (β = −0.027, SE = 0.016, *p*_*FDR*_ = 0.120) and clustering coefficient showed no significant changes over time (β = −0.001, SE = 0.016, *p*_*FDR*_ = 0.935). Sex did not have any significant effect on graph theory metrics (*p*_*FDR*_ > 0.05).

### 3.2 Latent change score modelling

Results of the SEM analyses are shown in Table 2. The models examining associations of szPGRS with white matter water diffusion MRI parameters fit the data well (white matter tract FA: RMSEA < 0.058, CFI > 0.940, SRMR < 0.030 and white matter tract MD: RMSEA < 0.075, CFI > 0.923, SRMR < 0.039). Associations between FA and szPGRS were non-significant for level or change in any tract (*p*_*FDR*_ > 0.05). Associations between MD and szPGRS were non-significant for level (*p*_*FDR*_ > 0.05). However, change in MD showed significant associations with szPGRS for the splenium (r = 0.132, *p*_*FDR*_ = 0.040), arcuate (r = 0.291, *p*_*FDR*_ = 0.040), ATR (r = 0.215, *p*_*FDR*_ = 0.040) and cingulum (r = 0.165, *p*_*FDR*_ = 0.040). Scatterplots of the relationship between the percentage of change in MD from significant associations in the SEM models (from 73 years to 76 years) and szPGRS at p ≤ 1.0 are presented in Supplementary Material Fig. 1. Results of the SEM analyses for FA and MD using szPGRS at P ≤ 0.1 and 0.5 thresholds are presented in Supplementary Material Tables 3 and 4.

**Table 2:** Structural equation modelling results. Standardised estimates from the associations between polygenic risk score for schizophrenia (szPGRS) at a threshold of P ≤ 1.0 and level and change in connectivity.

	Level (age 73)			Change (age 73 to 76)		
	*r*	*SE*	*p*_*FDR*_	*r*	*SE*	*p*_*FDR*_
FA						
Genu	0.039	0.040	0.674	−0.042	0.049	0.477
Splenium	−0.009	0.058	0.930	−0.082	0.063	0.266
Arcuate	0.021	0.003	0.930	−0.073	0.002	0.477
ATR	0.019	<0.001	0.930	−0.135	0.001	0.266
Cingulum	0.125	0.004	0.147	−0.268	0.004	0.266
Uncinate	0.061	0.002	0.674	−0.074	0.003	0.477
ILF	−0.005	0.003	0.930	−0.156	0.004	0.477
MD						
Genu	0.003	0.069	0.946	0.007	0.093	0.875
Splenium	−0.037	0.112	0.821	0.132	0.158	0.040*
Arcuate	0.007	<0.001	0.946	0.291	<0.001	0.040*
ATR	−0.035	<0.001	0.830	0.215	0.001	0.040*
Cingulum	−0.118	<0.001	0.098	0.165	<0.001	0.040*
Uncinate	−0.052	<0.001	0.821	0.024	0.001	0.704
ILF	−0.032	0.007	0.830	0.304	0.011	0.434
Connectome						
Mean edge weight	0.042	0.002	0.369	−0.039	0.001	0.551
Strength	0.037	0.038	0.369	−0.035	0.033	0.551
Global efficiency	0.039	0.001	0.369	−0.035	0.001	0.551
Clustering coefficient	0.040	0.001	0.369	−0.034	0.001	0.551

*Note:* SE: Standard error, FA: fractional anisotropy, MD: mean diffusivity, ATR: anterior thalamic radiations, ILF: inferior longitudinal fasciculus, *p-*values are corrected for multiple comparison using FDR. Asterisks represent significance (*p*_*FDR*_ < 0.05).

Models examining associations between the level and change of szPGRS and graph theory metrics showed excellent fit to the data (RMSEA < 0.029, CFI > 0.985, SRMR < 0.021). There were no significant associations between szPGRS and the baseline level of graph theoretical metrics (r < 0.042, *p*_*FDR*_ > 0.05) or with their 3-year change (r < −0.039, *p*_*FDR*_ > 0.05; Table 2). The addition of blood pressure as a covariate did not have any significant effect on the results of any of the SEM models described above (*p*_*FDR*_ > 0.05). Results of the SEM analyses for graph theory measures using szPGRS at P ≤ 0.1 and 0.5 thresholds are presented in Supplementary Material Tables 3 and 4.

**Fig. 5:**
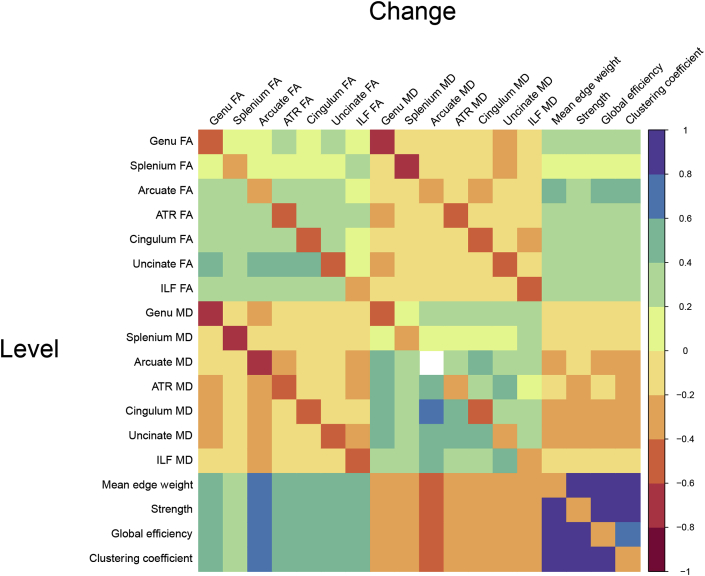
Heatmap illustrating Spearman's correlation coefficients for baseline level (age 73 years old, lower diagonal) and change (73–76 years old, upper diagonal) in white matter diffusion parameters and graph theory metrics. Diagonal coefficients represent the association between baseline and change for each metric derived from the SEM models described in Fig. 1. Individual slopes for change were derived from the SEM models. Blank cells denote those associations that did not survive multiple comparisons correction (*p*_FDR_ < 0.05). ATR = Anterior thalamic radiations; ILF = Inferior longitudinal fasciculus.

Associations between extracted slopes from the SEM models and baseline levels for FA, MD and graph theory metrics are presented in Figure 5. These results illustrate that changes are highly coupled within diffusion MRI parameters and graph theory measures for level (age 73) and longitudinal change (age 73 to age 76) for structural brain connectivity in older age. Diagonal coefficients show the associations between level and change for structural connectivity and indicates that participants with lower (‘healthier’) MD values show greater increases in MD, and those with higher (‘healthier’) FA values show steeper decreases in FA. Similarly, those with higher graph theoretical metrics at baseline showed steeper declines over time.

### 3.3 Mediation analysis

We aimed to identify mediation candidates that were consistent with the hypothesis that a higher genetic predisposition to schizophrenia is related to lower cognitive functions through the disruption of structural brain connectivity (for a detailed description of the model see Fig. 2). First of all, a model examining associations between szPGRS and g_*f*_ was computed which showed good fit to the data (RMSEA = 0.059, CFI = 0.935, SRMR = 0.049). There was a significant association between szPGRS and the baseline level of g_*f*_ (*r* = −0.145, *p* = 0.001) but not with 3-year change in g_*f*_ (*r* = 0.003, *p* = 0.962). Full results of associations between szPGRS and in baseline levels and changes g_*f*_ and MD are presented in Supplementary Material Table 5. Given we did not find any significant associations between szPGRS and level/change in g_*f*_ and MD, there were no plausible candidates for a mediation model.

## 4 Discussion

The present study found significant associations between a greater genetic risk for schizophrenia and longitudinal increases in MD in the splenium, arcuate, ATR and cingulum fasciculi over 3 years using the largest schizophrenia GWAS to date (Pardiñas et al., 2018) and an improved reference tract segmentation analysis (Muñoz Maniega et al., 2017). We did not find any significant associations between szPGRS and change in FA or graph theoretical metrics. The results of this investigation show that there were significant differences in the microstructure of most white matter tracts studied here and network topology over a short period of time in this older age cohort. Particularly, we found decreases in FA (standardised *r* from 0.056 to −0.062) in most white matter tracts and graph theory measures (standardised *r* from −0.001 to −0.039) as well as increases in MD (standardised *r* from 0.171 to 0.452) in all white matter tracts over this 3-year-old period.

Numerous studies have shown consistent structural brain alterations in patients with schizophrenia. These include reductions in both grey and white matter compared to healthy controls. However, cross-sectional studies analysing the effect of szPGRS on brain structure in non-clinical samples have not been conclusive (Van der Auwera et al., 2017, 2015). Ritchie et al. (2017) showed a significant positive longitudinal association between szPGRS - derived from a previous GWAS- and 3-year change in a general factor of tract-averaged MD in the sample used in the present study. However, a limitation of generating a general factor from water diffusion MRI parameters measured in multiple tracts is that it describes commonalities among white matter tracts while excluding tract-specific individualities. Our findings indicate that the association of szPGRS with white matter MD is strongly driven by the splenium, arcuate, ATR and cingulum, all tracts previously implicated in schizophrenia. Structural abnormalities in the corpus callosum in schizophrenia have been well documented affecting interhemispheric communication in patients (Foong et al., 2000; Woodruff et al., 1995). The arcuate fasciculus as an associative fibre connects the frontal cortex with the temporal and parietal cortices and may underlie language processing anomalies in the disorder (Abdul-Rahman et al., 2012). The ATR serves as a link between the thalamic nuclei and the prefrontal cortex, and dysfunction of the thalamus has been associated with the pathophysiology of schizophrenia, particularly with cognitive deficits and negative symptoms (Mamah et al., 2010). The cingulum is the most prominent white matter tract in the limbic system and has been previously reported to be impaired in schizophrenia (Fujiwara et al., 2007).

To our knowledge, there are no studies that have investigated the association between the structural connectome and genetic risk for schizophrenia; the fact that we did not find a significant effect of szPGRS on either the baseline level or change in structural brain connectivity (as measured by graph theoretical metrics) suggests that common genetic variants for schizophrenia and topological brain characteristics may not share a direct genetic mechanism. Nevertheless, szPGRS evinced non-significant detrimental relations with all brain structural metrics. The fact that the LBC1936 comprises relatively healthy, community-dwelling older adults, none of whom have schizophrenia, coupled with the relatively brief (3 year) period of follow-up may have limited our ability to detect slighter effects. Interestingly, a previous study on targeted genetic analysis showed that differentially expressed genes in a well-characterised rat model of vascular white matter disease were associated with white matter hyperintensities (which exhibit elevated MD and reduced FA) in the LBC1936 and these included genes associated with schizophrenia and neurodevelopmental intellectual disabilities (Lopez et al., 2015). These results suggest that genetic risk for schizophrenia may have a role in age-related changes in brain structural connectivity, even among individuals who are not diagnosed with schizophrenia. Previous studies have suggested the conceptualization of schizophrenia as a syndrome of accelerated ageing (Kirkpatrick et al., 2008) indicating, for instance, significant declines in white matter coherence more than twice that of age-matched controls (Kochunov et al., 2013), with this reduction being linear from early adulthood and steeper as a function of increasing age (Cropley et al., 2017). Therefore, it may be possible that higher szPGRS confers certain risks for accelerated white matter ageing in healthy older participants. It is also likely that other factors such as gene-gene interactions, rare variants, and gene-environment interplay may help to explain the association between risk variants for schizophrenia and brain structural impairments (Van der Auwera et al., 2017).

In general, white matter tracts showed reductions in FA (standardised *r* from 0.056 to −0.062) and increases in MD (standardised *r* from 0.171 to 0.452) as a function of increasing age. These results are in line with those of previous studies where white matter microstructure declines with age (reviewed in Bennett and Madden, 2014). For instance, we found that MD of more frontal white matter tracts was more affected while more occipital tracts were more resilient to the effects of age (see Table 1). This is consistent with the hypothesis that ageing has region-specific effects, in particular the existence of an anterior-posterior gradient of age-related decline whereby tracts that are the last to develop are the most vulnerable to the ageing process (Bennett and Madden, 2014; Cox et al., 2016; Qiu et al., 2015). This pattern could be a consequence of the finding that later developed tracts are more thinly myelinated and therefore more susceptible to decline (Bartzokis et al., 2004). The ATR and uncinate fasciculi, conversely, showed an increase in FA with age in this study. White matter fibres within these tracts are known to have a complex architecture due to the presence of a large number of crossing fibres (Niida et al., 2013; Olson et al., 2015). Since FA is highly dependent on white matter architecture (Pierpaoli et al., 1996), it is possible that a loss of white matter fibres might lead to an increase in FA if the remaining fibres are more uniformly orientated than they were previously (Jones et al., 2006). Therefore, the observed increase of FA in the ATR and uncinate fasciculi may reflect the overall effect of loss of crossing fibres resulting from age-related neurodegeneration. This combination of observations provides some support for the conceptual premise that diffusion MRI parameters are significantly associated with cognitive decline in ageing cohorts (Madden et al., 2012) as well as in patients diagnosed with schizophrenia (Alloza et al., 2016; Kochunov et al., 2017).

This study found that those participants with ‘healthier’ white matter at baseline showed a steeper decline over time (see Figure 5). This same pattern for other brain imaging parameters has previously been reported in this sample and has been suggested to represent the Law of Initial Value and regression to the mean (Ritchie et al., 2015; Wilder, 1957), indicating that there may be more neurobiological processes that can affect those with ‘healthier’ white matter at baseline than those with a less healthy white matter. Given that there were no significant associations between szPGRS and baseline white matter measures in this study, it is perfectly reasonable for the associations between szPGRS and change in MD, and between baseline level of MD and change in MD to be non-coincidental phenomena – that is, for the common variance between szPGRS and change, and between baseline and change, to be mutually exclusive.

As an additional analysis we tested whether change in MD would mediate the association between szPGRS and change in fluid intelligence. We found significant negative associations between baseline levels of MD in the splenium, arcuate and ATR and baseline levels of g_*f*_ as well as a significant negative association between szPGRS and baseline g_*f*_. These results indicate that higher baseline g_*f*_ is associated with a ‘healthier’ baseline white matter microstructure in this cohort. However, we did not find an association between szPGRS and change in g_*f*_ and thus, the data did not support the hypothesis that these candidates were plausible for a mediation model. It is likely that the relatively brief (3 year) period of follow-up may have limited our ability to detect modest effects, indicating that longer follow-ups and potentially the study of other factors that contribute to cognitive decline in older age, may be required. Ritchie et al. (2015) reported significant associations between change in FA and change in fluid intelligence, indicating that MD of the white matter tracts studied here may be more pertinent to other cognitive functions. Further work is required to investigate this hypothesis. Therefore, these data show that szPGRS is related to some selective MD changes over time, but not to cognitive decline over this same period.

This study is one of the first to examine the ageing of the human structural connectome longitudinally from healthy older participants. By taking a longitudinal approach, our results shed light on age-related brain structural decline by minimizing problems inherent to cross-sectional mediation methods (Hofer and Sliwinski, 2001; Lindenberger et al., 2011) while allowing age-related changes and associations with genetic risk factors to be investigated independently of age. The current study found subtle decreases in all graph theory metrics over a period of three years. Mean edge weight and strength decreased significantly over time while decreases in global efficiency and clustering coefficient did not reach significance. Reductions in graph theory measures, which describe topological aspects of the brain's networks were found to co-exist with microstructural declines in white matter tracts over time as shown in Figure 5. These results are consistent with the modest pre-existent literature on structural connectivity in ageing populations (Damoiseaux, 2017). In a cross-sectional study, Gong et al. (2009) reported lower overall connectivity and local efficiency as a function of age, but no differences in global efficiency. Zhao et al. (2015) using streamline density as a weighted measure, found an inverted U-shape for strength and global efficiency and a U-shape trajectory for clustering coefficient across the lifespan. This latter finding may be able to explain the nominal change in clustering coefficient in our study. Moreover, functional and structural connectivity studies seem to show closely related differences associated with age (Betzel et al., 2014; Fjell et al., 2016; Zimmermann et al., 2016).

### 4.1 Limitations

The generalisability of these results is subject to certain limitations. For instance, this study only covered a period of three years, which may not be sufficient to capture the effect of more subtle age-related changes. Measurement across only two occasions precludes consideration on non-linear trends or accelerating changes as a function of genetic liability to schizophrenia. Likewise, as sample sizes increases for GWAS better predictive power will be achieved by szPGRS. The choice of the most liberal SNP inclusion threshold (all SNPs, *p* = 1.00) may have affected the results presented here; however, this threshold has been recommended previously in order to allow replication by other studies and to maximise the potential predictive capacity (Ware et al., 2017). Furthermore, we present results for the SEM analysis at p ≤ 0.1 and p ≤ 0.5 szPGRS thresholds in Supplementary Material Tables 3 and 4.

For tractography, we extracted water diffusivity MRI parameters from twelve major white matter tracts, overlooking the rest of the connections. However, these tracts were well-characterised and reliably measured as previously reported (Bastin et al., 2010; Muñoz Maniega et al., 2017); moreover, we took account of all these connections by calculating whole-brain mean edge weight to include mean FA of all connections identified in the structural connectome. We also acknowledge the possibility that tract measures of FA and MD could potentially be affected by partial volume effects (pve) of cerebrospinal fluid (CSF). However, in the current analysis we segmented the tracts of interest using probabilistic neighbourhood tractography, which uses single seed point tractography, followed up by a streamline rejection criterion where individual streamlines are retained or rejected based on their probabilities under the topology model (Clayden et al., 2009a). This results in a tract made up from a ‘core’ of the streamlines that follow the expected tract topology, which is potentially less sensitive to pve than other tractography methods which segment larger white matter regions. In addition, we calculated tract-averaged MD and FA values weighted by the connection probability, which is usually lower at the edges of the tract, with the result that white matter voxels closer to CSF structures would have lower contribution to the mean.

The global metrics calculated across the entire structural connectome cannot address the possibility that specific networks (i.e. subsets of nodes or edges) show age-related changes that are more sensitive to szPGRS. In addition, network comparability issues may arise as a result of differing density between networks since the number of nodes or network's degree influences the computation of global theory metrics (see Brain Connectivity Toolbox for a detailed description of metrics: https://sites.google.com/site/bctnet). Therefore, we chose to control each subject's graph theory measure for edge density. The validity of the correction of density remains an issue in need of further exploration. For instance, correcting for density may affect regression coefficients due to the apparent multicollinearity between graph theory metrics. Further limitations inherent to longitudinal studies include attrition and loss of follow-up. However, we implemented maximum likelihood estimation methods that reduce missing data bias derived from longitudinal attrition. Finally, we implemented latent change score models across all parameters, including those in which we only had a single indicator (graph theoretical and callosal metrics). We did so to maintain comparability of analytic approach and results across all analyses, but the single indicator change score models should essentially be considered difference scores because they are unable to parse out error variance (Gollwitzer et al., 2014).

Finally, further research is required to examine whether any of the associations between water diffusion metrics and szPGRS are sex-specific, or alternatively show similar patterns in males and females. Recently, a growing number of studies have suggested a reduced leftward structural asymmetry in schizophrenia compared to healthy controls (Ribolsi et al., 2014), hence in this study we did not constrain the loadings to be equal for the left and right white matter tracts in the SEM analysis. However, further research is needed to address in greater detail this hypothesis.

## 5 Conclusions

The present longitudinal study was designed to determine the association of genetic risk for schizophrenia with brain structure. We found a significant association between higher szPGRS and increasing MD for the splenium, arcuate, ATR and cingulum, consistent with the hypothesis that higher genetic liability for schizophrenia is related to accelerated brain ageing among relatively healthy older adults. We also present some valuable data on the nature of brain connectivity changes in older age. Over a three-year-old period we found significant differences in white matter microstructure for a range of major white matter tracts; for most of these tracts we reported significant age-related decreases in FA and increases in MD. This decline in white matter microstructure was accompanied by disruptions at the topological level. All graph theory metrics showed subtle decreases over this narrow timeframe. However, only mean edge weight and strength reached our specified significance level. In this study we also examined the hypothesis that higher szPGRS is associated with a steeper decline in cognition via change in white matter structure in older age. Significant negative associations between baseline levels of general fluid intelligence and szPGRS and baseline levels of MD in the splenium, arcuate and ATR were found. Taken together, these findings suggest subtle age-related declines in white matter connectivity which take place over a relatively short period of time in older age, with szPGRS conferring some risk for these changes in brain structure.
