# Supplementary material for: Polygenic risk score for schizophrenia and structural brain connectivity in older age: A longitudinal connectome and tractography study
Source: Neuroimage. 2018 Dec;183:884–96. doi: 10.1016/j.neuroimage.2018.08.075 (PMC6215331; doi:10.1016/j.neuroimage.2018.08.075)
Supplement: Supplementary Material [file mmc1.docx]

**Supplementary Material Table 1:** Descriptive statistics of individual cognitive tests from the Wechsler Adult Scale - III across both waves (age 73 and age 76).

|  | *n* | Age 73 | *n* | Age 76 | *r* | *SE* | *p_FDR_* |
| --- | --- | --- | --- | --- | --- | --- | --- |
| **Wechsler Adult Scale - III** |  |  |  |  |  |  |  |
| Matrix reasoning | 690 | 13.37 (4.90) | 574 | 13.16 (5.00) | -0.050 | 0.019 | 0.013* |
| Letter number sequencing | 690 | 10.98 (3.02) | 573 | 10.49 (2.98) | -0.122 | 0.019 | < 0.001* |
| Block Design | 690 | 34.07 (10.07) | 577 | 32.43 (10.15) | -0.010 | 0.016 | < 0.001* |
| Symbol search | 689 | 24.63 (6.19) | 573 | 24.53 (6.58) | -0.046 | 0.018 | 0.013* |
| Digit symbol | 689 | 56.24 (12.27) | 570 | 53.73 (12.78) | -0.156 | 0.013 | < 0.001* |
| Digit span backwards | 692 | 7.84 (2.33) | 581 | 7.76 (2.36) | -0.032 | 0.018 | 0.075 |

*Note:* SD: Standard deviation, r: standardised estimates from the linear mixed models, SE: standard error. Asterisks represent significance from the linear mixed models (*p_FDR_* < 0.05).

**Supplementary Material Table 2:** Percentage of health conditions across both waves (age 73 and age 76).

| **Health** | Age 73 | Age 76 |
| --- | --- | --- |
| Diabetes | 10.71% | 12.03% |
| Hypertension | 48.81% | 54.22% |
| High cholesterol | 41.52% | 47.68% |
| History of cardiovascular disease | 27.23% | 33.34% |

**Supplementary Material Figure 1:** Scatterplot of the relationship between the percentage of change in MD (mean of left-right values from 73 years to 76 years) and szPGRS at p ≤ 1.0. Standardised linear regression coefficients (*β* ) were derived from the regression between percentage of change in MD and residuals from szPGRS with MDS components and sex. Note that these plots and the accompanying betas (using complete data only) correspond well with the SEM-implied significant associations reported in the main manuscript which account for missingness (FIML) and treat variables and their change as latent (it is inadvisable to extract factor scores directly from the SEM due to issues of factor score indeterminacy, e.g. Grice, 2001). Black line represents linear regression and in grey the 95% confidence interval.

*β* = 0.162, *p =* 0.001

*β* = 0.133, *p =* 0.014

*β* = 0.122, *p =* 0.019

*β* = 0.106, *p =* 0.049

**Supplementary Material Table 3:** Structural equation modelling results. Standardised estimates from the associations between polygenic risk score for schizophrenia (szPGRS) at a threshold of P ≤ 0.1 and level and change in connectivity.

|  | Threshold of P ≤ 0.1 | | | | | |
| --- | --- | --- | --- | --- | --- | --- |
|  | Level (age 73) | | | Change (age 73 to 76) | | |
|  | *r* | *SE* | *p_FDR_* | *r* | *SE* | *p_FDR_* |
| **FA** |  |  |  |  |  |  |
| Genu | 0.021 | 0.153 | 0.899 | -0.037 | 0.156 | 0.664 |
| Splenium | -0.004 | 0.209 | 0.935 | -0.077 | 0.196 | 0.458 |
| Arcuate | -0.023 | 0.011 | 0.899 | -0.046 | 0.007 | 0.689 |
| ATR | 0.017 | 0.008 | 0.899 | -0.098 | 0.008 | 0.572 |
| Cingulum | 0.122 | 0.014 | 0.385 | -0.230 | 0.013 | 0.458 |
| Uncinate | 0.063 | 0.009 | 0.899 | -0.066 | 0.009 | 0.664 |
| ILF | -0.048 | 0.011 | 0.899 | -0.173 | 0.011 | 0.689 |
|  |  |  |  |  |  |  |
| **MD** |  |  |  |  |  |  |
| Genu | 0.045 | 0.263 | 0.526 | -0.01 | 0.28 | 0.847 |
| Splenium | -0.047 | 0.431 | 0.526 | 0.116 | 0.507 | 0.079 ∙ |
| Arcuate | -0.008 | 0.001 | 0.873 | 0.207 | 0.001 | 0.079 ∙ |
| ATR | -0.063 | 0.002 | 0.526 | 0.173 | 0.002 | 0.115 |
| Cingulum | -0.124 | 0.001 | 0.231 | 0.189 | 0.001 | 0.079 ∙ |
| Uncinate | -0.094 | 0.001 | 0.339 | 0.025 | 0.001 | 0.734 |
| ILF | 0.022 | 0.023 | 0.873 | 0.076 | 0.029 | 0.734 |
|  |  |  |  |  |  |  |
| **Connectome** |  |  |  |  |  |  |
| Mean edge weight | 0.035 | 0.006 | 0.537 | -0.014 | 0.004 | 0.862 |
| Strength | 0.031 | 0.132 | 0.537 | -0.010 | 0.100 | 0.862 |
| Global efficiency | 0.035 | 0.004 | 0.537 | -0.013 | 0.003 | 0.862 |
| Clustering coefficient | 0.031 | 0.004 | 0.537 | -0.012 | 0.003 | 0.862 |

*Note:* SE: Standard error, FA: fractional anisotropy, MD: mean diffusivity, ATR: anterior thalamic radiations, ILF: inferior longitudinal fasciculus, *p-*values are corrected for multiple comparison using FDR. Dots tendency towards significance.

**Supplementary Material Table 4:** Structural equation modelling results. Standardised estimates from the associations between polygenic risk score for schizophrenia (szPGRS) at a threshold of P ≤ 0.5 and level and change in connectivity.

|  | Threshold of P ≤ 0.5 | | | | | |
| --- | --- | --- | --- | --- | --- | --- |
|  | Level (age 73) | | | Change (age 73 to 76) | | |
|  | *r* | *SE* | *p_FDR_* | *r* | *SE* | *p_FDR_* |
| **FA** |  |  |  |  |  |  |
| Genu | 0.032 | 0.071 | 0.868 | -0.036 | 0.072 | 0.583 |
| Splenium | -0.008 | 0.097 | 0.868 | -0.97 | 0.091 | 0.175 |
| Arcuate | 0.010 | 0.005 | 0.868 | -0.060 | 0.003 | 0.596 |
| ATR | 0.051 | 0.004 | 0.868 | -0.150 | 0.004 | 0.175 |
| Cingulum | 0.110 | 0.006 | 0.581 | -0.239 | 0.006 | 0.175 |
| Uncinate | 0.055 | 0.004 | 0.868 | -0.056 | 0.004 | 0.583 |
| ILF | -0.025 | 0.005 | 0.868 | -0.322 | 0.005 | 0.583 |
|  |  |  |  |  |  |  |
| **MD** |  |  |  |  |  |  |
| Genu | 0.034 | 0.122 | 0.583 | -0.018 | 0.129 | 0.730 |
| Splenium | -0.055 | 0.200 | 0.465 | 0.152 | 0.235 | 0.021* |
| Arcuate | -0.041 | 0.001 | 0.583 | 0.212 | < 0.001 | 0.059 ∙ |
| ATR | -0.098 | 0.001 | 0.324 | 0.230 | 0.001 | 0.052 ∙ |
| Cingulum | -0.115 | 0.001 | 0.324 | 0.160 | 0.001 | 0.059 ∙ |
| Uncinate | -0.084 | 0.001 | 0.324 | 0.030 | 0.001 | 0.581 |
| ILF | 0.013 | 0.011 | 0.866 | 0.149 | 0.013 | 0.477 |
|  |  |  |  |  |  |  |
| **Connectome** |  |  |  |  |  |  |
| Mean edge weight | 0.065 | 0.003 | 0.233 | -0.046 | 0.002 | 0.460 |
| Strength | 0.060 | 0.061 | 0.233 | -0.041 | 0.046 | 0.460 |
| Global efficiency | 0.061 | 0.002 | 0.233 | -0.042 | 0.001 | 0.460 |
| Clustering coefficient | 0.063 | 0.002 | 0.233 | -0.044 | 0.001 | 0.460 |

*Note:* SE: Standard error, FA: fractional anisotropy, MD: mean diffusivity, ATR: anterior thalamic radiations, ILF: inferior longitudinal fasciculus, *p-*values are corrected for multiple comparison using FDR. Asterisks represent significance (*p_FDR_* < 0.05) and dots tendency towards significance.

**Supplementary Material Table 5:** Structural equation modelling results. Standardised estimates from the associations between polygenic risk score for schizophrenia (szPGRS) at a threshold of P ≤ 1.0 and level and change in white matter microstructure and in general fluid intelligence.

| Path type | Path | *r* | *SE* | *p_FDR_* |
| --- | --- | --- | --- | --- |
| Level - Level | szPGRS- g*_f_* | -0.145 | 0.029 | 0.001* |
|  | Splenium MD - g*_f_* | -0.113 | 0.032 | 0.020* |
|  | Arcuate MD - g*_f_* | -0.132 | 0.032 | 0.012* |
|  | ATR MD - g*_f_* | -0.115 | 0.028 | 0.044* |
|  | Cingulum MD - g*_f_* | -0.067 | 0.030 | 0.184 |
|  |  |  |  |  |
| Level - Change (Δ) | szPGRS- Δ g*_f_* | 0.003 | 0.015 | 0.962 |
|  | Splenium MD - Δ g*_f_* | -0.129 | 0.017 | 0.122 |
|  | Arcuate MD - Δ g*_f_* | -0.112 | 0.018 | 0.176 |
|  | ATR MD - Δ g*_f_* | -0.171 | 0.016 | 0.122 |
|  | Cingulum MD - Δ g*_f_* | -0.109 | 0.017 | 0.183 |
|  | g*_f_* - Δ Splenium MD | 0.057 | 0.036 | 0.264 |
|  | g*_f_* - Δ Arcuate MD | -0.130 | 0.022 | 0.134 |
|  | g*_f_* - Δ ATR MD | -0.261 | 0.029 | 0.012* |
|  | g*_f_* - Δ Cingulum MD | -0.090 | 0.033 | 0.212 |
|  |  |  |  |  |
| Change (Δ) – Change (Δ) | Δ g*_f_* - Δ Splenium MD | 0.026 | 0.295 | 0.916 |
|  | Δ g*_f_* - Δ Arcuate MD | -0.021 | 0.186 | 0.916 |
|  | Δ g*_f_* - Δ ATR MD | 0.051 | 0.279 | 0.916 |
|  | Δ g*_f_* - Δ Cingulum MD | -0.010 | 0.274 | 0.916 |

*Note:* SE: Standard error, g*_f_* : general fluid intelligence, MD: mean diffusivity, ATR: anterior thalamic radiations, *p-*values are corrected for multiple comparison using FDR. Asterisks represent significance (*p_FDR_* < 0.05).

**References**

Grice, J.W., 2001. Computing and evaluating factor scores. Psychol. Methods 6, 430–450.
